# SUMOylated MAFB promotes colorectal cancer tumorigenesis

**DOI:** 10.18632/oncotarget.13129

**Published:** 2016-11-05

**Authors:** Lin-Sen Yang, Xiao-Jian Zhang, Yin-Yin Xie, Xiao-Jian Sun, Ren Zhao, Qiu-Hua Huang

**Affiliations:** ^1^ State Key Laboratory of Medical Genomics, Rui Jin Hospital, Shanghai Jiao Tong University School of Medicine, Shanghai 200025, China; ^2^ Department of General Surgery, Rui Jin Hospital, Shanghai Jiao Tong University School of Medicine, Shanghai 200025, China

**Keywords:** MAFB, SUMOylation, cell cycle, CDK6, colorectal cancer

## Abstract

The transcription factor, v-maf avian musculoaponeurotic fibrosarcoma oncogene homolog B (MAFB), promotes tumorigenesis in some cancers. In this study, we found that MAFB levels were increased in clinical colorectal cancer (CRC) samples, and higher expression correlated with more advanced TNM stage. We identified *MAFB* amplifications in a majority of tumor types in an assessment of The Cancer Genome Atlas database. Altered *MAFB* levels due to gene amplification, deletion, mutation, or transcription upregulation occurred in 9% of CRC cases within the database. shRNA knockdown experiments demonstrated that MAFB deficiency blocked CRC cell proliferation by arresting the cell cycle at G0/G1 phase *in vitro*. We found that MAFB could be SUMOylated by SUMO1 at lysine 32, and this modification was critical for cell cycle regulation by MAFB in CRC cells. SUMOylated MAFB directly regulated cyclin-dependent kinase 6 transcription by binding to its promoter. MAFB knockdown CRC cell xenograft tumors in mice grew more slowly than controls, and wild-type MAFB-overexpressing tumors grew more quickly than tumors overexpressing MAFB mutated at lysine 32. These data suggest that SUMOylated MAFB promotes CRC tumorigenesis through cell cycle regulation. MAFB and its SUMOylation process may serve as novel therapeutic targets for CRC treatment.

## INTRODUCTION

In spite of advancements in treatment options, including surgical resection, radiotherapy, immunotherapy and chemotherapy, colorectal cancer (CRC) remains the third most common cancer globally, with the fourth highest mortality rate [1, 2]. About 50% percent of CRC patients experience multiple relapses and die within five years of diagnosis [3]. The tumor node metastasis (TNM) staging system is currently the most important guideline in CRC therapeutic regimen selection and prognosis prediction [4]. However, patients with the same CRC stage may have different pathological processes and prognoses. To improve upon existing treatment and prognostic options, the molecular mechanisms driving CRC must be better understood. Additionally, specific CRC biomarkers would allow for more accurate predictions of patient responses to specific treatments, potentially improving survival outcomes [5].

V-maf avian musculoaponeurotic fibrosarcoma oncogene homolog B (MAFB) belongs to the Maf transcription factor family that contains N-terminal transactivation and C-terminal basic leucine-zipper (bZip) deoxyribonucleic acid (DNA) binding domains [6]. As a transcription factor, MAFB is well known for its roles in development and differentiation of many organs, tissues and cells, including pancreas development [7], macrophage differentiation [8], osteoclastogenesis [9], and hindbrain segmentation [10]. MAFB aberrations often lead to disease; chromosomal translocations result in ectopic MAFB expression in human myeloma cells [11], domain-specific *MAFB* mutations cause multicentric carpotarsal osteolysis syndrome [12], and elevated MAFB expression was observed in acute myeloid leukemia blasts [13]. Yang, *et al.* [14] found that miR-223 suppresses nasopharyngeal carcinoma cell proliferation and migration by targeting *MAFB* mRNA. Lu, *et al.* [15] reported that MafB overexpression enhanced cell foci formation and increased cyclin B1 and D2 expression. MafB downregulation reduced BrdU incorporation in the same insulinoma cell lines. These findings suggest that MAFB overexpression may promote tumor progression.

Mouse MafB is modified by SUMO (small ubiquitin-like modifier), and this modification is critical for its transcriptional activities regulating macrophage differentiation [16]. However, it was still unclear whether human MAFB could be SUMOylated. Ninety-seven percent of the human MAFB protein-coding sequence is identical to that of mouse MafB [7], suggesting that human MAFB may also be SUMOylated. SUMOylation regulates subcellular localization, protein-DNA binding, protein-protein interactions, transcriptional regulation, DNA repair, and genome organization [17, 18], and also plays important roles in carcinogenesis [19]. Elucidating the potential roles of MAFB SUMOylation may prove useful for MAFB-related disease treatment.

In this study, cBioPortal was used as an online analytical tool to analyze *MAFB* aberrations in The Cancer Genome Atlas (TCGA) database. We also assessed *MAFB* expression in CRC specimens, and analyzed its association with tumor stage. Finally, we evaluated whether human MAFB is modified by SUMO1, and explored the specific role of MAFB in CRC progression.

## RESULTS

### TCGA data mining revealed aberrant *MAFB* amplification in CRC

**Figure 1:**
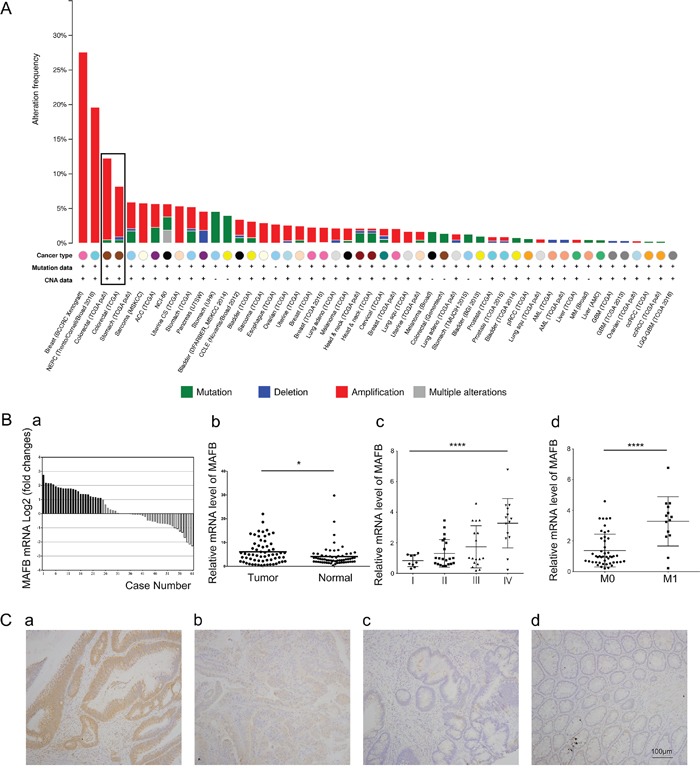
MAFB is upregulated in CRC Somatic MAFB alterations reproduced from TCGA database showing MAFB gene amplification in a majority of tumor types **A.** Altered MAFB levels occurred in 9% of CRC cases. MAFB was upregulated in CRC tissues **B.** MAFB levels in 61 paired CRC and matched adjacent non-tumor tissues were determined by RT-qPCR and normalized to *GAPDH*. Data are expressed as the log_2_ fold change (ΔCt [CRC/Non.]), and significant MAFB upregulation was defined as a log_2_ fold change >1 **Ba.** MAFB mRNA levels in: CRC and adjacent non-tumor tissues **Bb.** different stage CRC tissues **Bc.** and CRCs with and without metastases **Bd.** MAFB immunohistochemical staining in human CRC tissues and paired adjacent non-tumor tissues **C.** Strong positive MAFB expression in CRC **Ca.** weak positive MAFB expression in CRC **Cb.** negative MAFB expression in CRC **Cc.** and negative MAFB expression in non-tumor colorectal tissue **Cd.** bars: 100μm. Data are presented as means ± SD. *N.S.* (non-significant), *P*≥0.05; *P<0.05; ****P<0.0001.

Chromosome translocation in multiple myeloma results in aberrant MAFB expression [11, 20], and miR-223 suppresses nasopharyngeal carcinoma cell proliferation and migration by targeting MAFB [14]. We studied the status of MAFB in TCGA and found aberrant *MAFB* amplification in a majority of enrolled tumor types (Figure 1A). Altered *MAFB* levels due to gene amplification, deletion, mutation, or transcription upregulation occurred in 9% of CRC cases.

### MAFB was highly expressed in CRC tissues and correlated with pathological stage

Real-time quantitative polymerase chain reaction (RT-qPCR) was performed to assess MAFB expression in CRC tissues and paired adjacent non-tumor tissues from 61 surgically treated patients. Clinical pathological parameters, like age, gender, histological type and tumor location, did not correlate with *MAFB* level (Table 1). We found that *MAFB* was upregulated in 39.34% of CRC specimens, and was downregulated in 13.11% (Figure 1Ba). *MAFB* expression was increased in CRC samples as compared to normal tissues (p<0.05, Figure 1Bb). Differences in measured MAFB levels between TCGA and our clinical specimens may due to sample size variations. Increased *MAFB* expression was correlated with advanced tumor stages (Figure 1Bc, Table 1). The highest *MAFB* levels were measured in stage IV CRCs, while the lowest levels were in stage I CRCs (p<0.0001). A similar trend was observed in a comparison of metastatic and non-metastatic CRCs (p<0.0001) (Figure 1Bd, Table 1). Immunohistochemical staining showed that MAFB protein levels were also increased in CRC tissues as compared to adjacent non-tumor tissues (Figure 1Ca –1Cd).

**Table 1:** Correlation of MAFB expression with CRC patient clinicopathological parameters

Parameters	Number of cases (%)	MAFB expression		χ^2^	P-value
		Upregulation	Downregulation		
Age (years)					
<60	24(39.3%)	13	11	0.393622	0.530402
≥60	37(60.7%)	17	20		
Gender					
Male	34(55.7%)	16	18	0.138328	0.709949
Female	27(44.3%)	14	13		
Histological type					
Tubular adenocarcinoma	56(91.8%)	28	28	0.184961	0.667144
Mucinous adenocarcinoma	5(8.2%)	2	3		
Tumor location		22	19	2.296307	0.317222
Rectum and sigmoid	41(67.2%)	6	6		
Right colon	12(19.7%)	2	6		
Left colon	8(13.1%)				
TNM stage					
I	9(14.8%)	4	5	6.713018	0.009571
II	21(34.4%)	7	14		
III	17(27.9%)	6	11		
IV	14(22.9%)	13	1		
pT					
T1	3(4.9%)	1	2	2.623715	0.105278
T2	12(19.7%)	7	5		
T3	21(34.4%)	14	7		
T4	25(41.0%)	8	17		
pN					
N0	39(63.9%)	19	20	0.138751	0.709526
N1	9(14.8%)	3	6		
N2	13(21.3%)	8	5		
pM					
M0	47(77.0%)	17	30	13.868793	0.000196
M1	14(23.0%)	13	1		

### MAFB knockdown suppresses CRC cell proliferation

**Figure 2:**
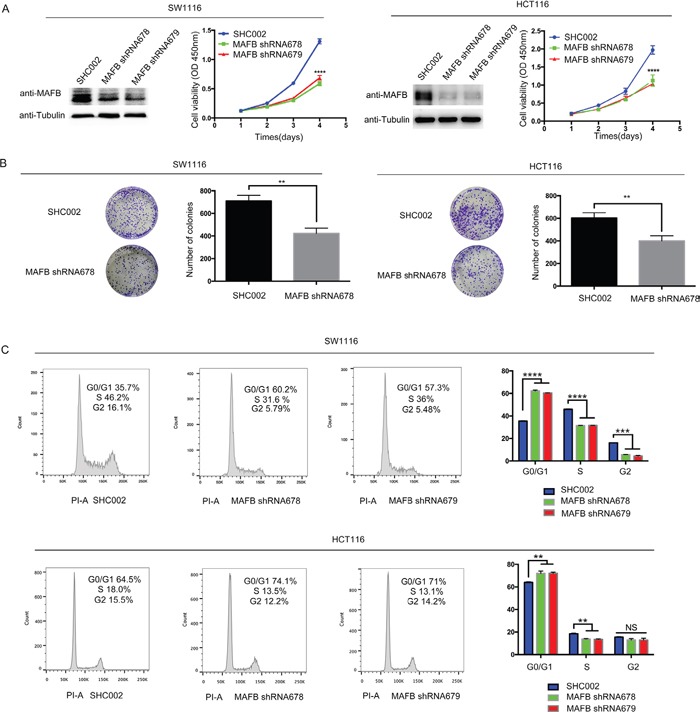
MAFB knockdown suppresses CRC cell proliferation through cell cycle dysregulation SW1116 and HCT116 cells were sorted by flow cytometry after infection with MAFB shRNA678, MAFB shRNA679 or scramble shRNA (SHC002) **A.** MAFB was detected by western blotting (WB), with tubulin as an internal control. Cell viability was indicated by absorbance at OD 450nm two h after CCK8 was added. Colony formation assay with SW1116 and HCT116 cells infected with MAFB shRNA678 or scramble shRNA (SHC002) **B.** Cells were cultured for 10 d and stained with crystal violet. Colonies were counted and analyzed. Cell cycle analysis of scramble shRNA (SHC002), MAFB shRNA678 and MAFB shRNA679 knockdown SW1116 cells by flow cytometry, showing the percentage of G0/G1 or S phase cells (upper panel) **C.** HCT116 and SW1116 results were the same (lower panel). Data are presented as means ± SEM. *P<0.05; **P<0.01; ***P<0.001; ****P<0.0001.

To address the pathological role of MAFB in CRC cells, a loss of function assay was performed by infecting the CRC cell line, SW1116, and HCT116 cells (endogenous *MAFB* expression patterns shown in Supplementary Figure S1) with lentivirus containing either shRNA targeting MAFB (shRNA678/shRNA679) or scramble shRNA (SHC002). Western blotting results showed that MAFB protein was effectively knocked-down (Figure 2A). MAFB knockdown cells showed reduced viability compared with scramble shRNA-transfected lines (Figure 2A). Similarly, colony-forming assays showed that MAFB knockdown cells formed fewer colonies than controls (Figure 2B).

### MAFB is critical for cell cycle regulation in CRC cells

Cell cycle progression and apoptosis were examined by flow cytometry analysis, which showed that MAFB knockdown dramatically increased the percentage of G0/G1 phase cells and decreased that of S phase cells (Figure 2C), but did not affect cell apoptosis (Supplementary Figure S2). We inferred that MAFB may regulate the G1/S phase transition and thereby promote CRC cell proliferation.

**Figure 3:**
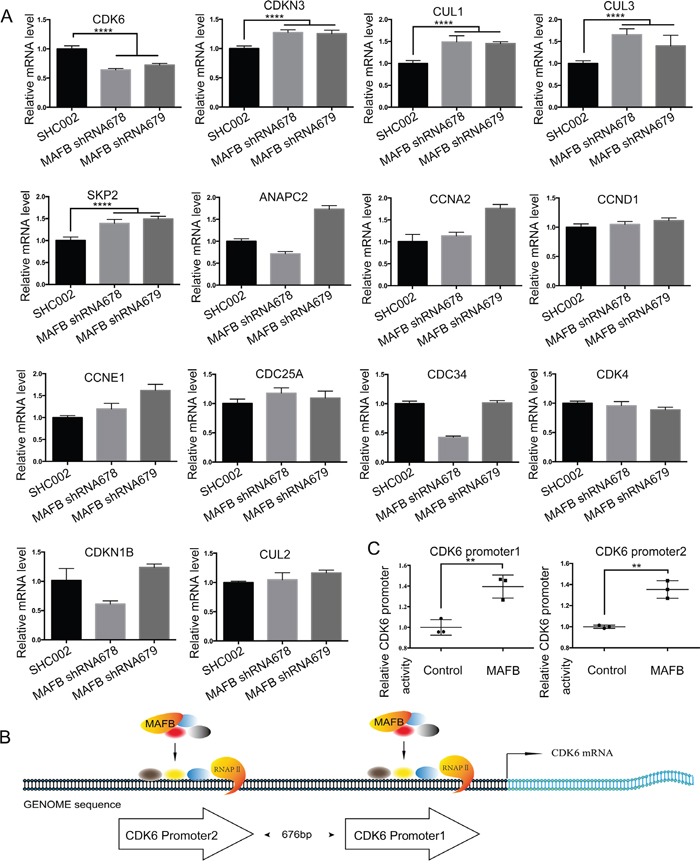
MAFB regulates expression of cell cycle factors RNA was extracted from SW1116 cells transfected with scramble shRNA (SHC002), MAFB shRNA678 or MAFB shRNA679 and cell cycle factors controlling the G1/S phase transition were analyzed via RT-qPCR **A**. Schematic of the human CDK6 promoter regions **B.** Relative CDK6 promoter activity was analyzed by luciferase assay **C.** The pRL-SV40 vector and pGL4.15 luciferase reporter vector containing the CDK6 promoter region were co-transfected with an MAFB expression vector or mock vector into 293T cells. Data are presented as means ± SEM. **P<0.01.

Murine MafB reportedly promotes cell proliferation with detectable changes in cell cycle factors [15]. As the percentage of S phase CRC cells was reduced following MAFB knockdown in our study, we speculated that MAFB may play important roles in regulating the expression of some cell cycle factors. We analyzed a series of cell cycle factors that control the G1/S phase transition via RT-qPCR. CDK6 expression was downregulated in MAFB knockdown SW1116 cells, while CDKN3, CUL1 and CUL3 were upregulated (Figure 3A). This was consistent with the findings of other G1 phase arrest studies [21–24]. We also found that CCNB1 and CCND1 were downregulated (Supplementary Figure S3), which was in agreement with previous work [15]. Only CDK6 downregulation corresponded with MAFB knockdown, indicating that CDK6 may be a direct target of MAFB. To investigate the regulatory role of MAFB on CDK6 expression, we constructed native CDK6 promoters to drive luciferase expression in a reporter gene assay (Figure 3B). Indeed, MAFB overexpression enhanced CDK6 promoter activity (Figure 3C). These data suggested that MAFB might promote CRC cell proliferation by regulating cell cycle factor levels.

### SUMO1 modified human MAFB in HEK293T cells

**Figure 4:**
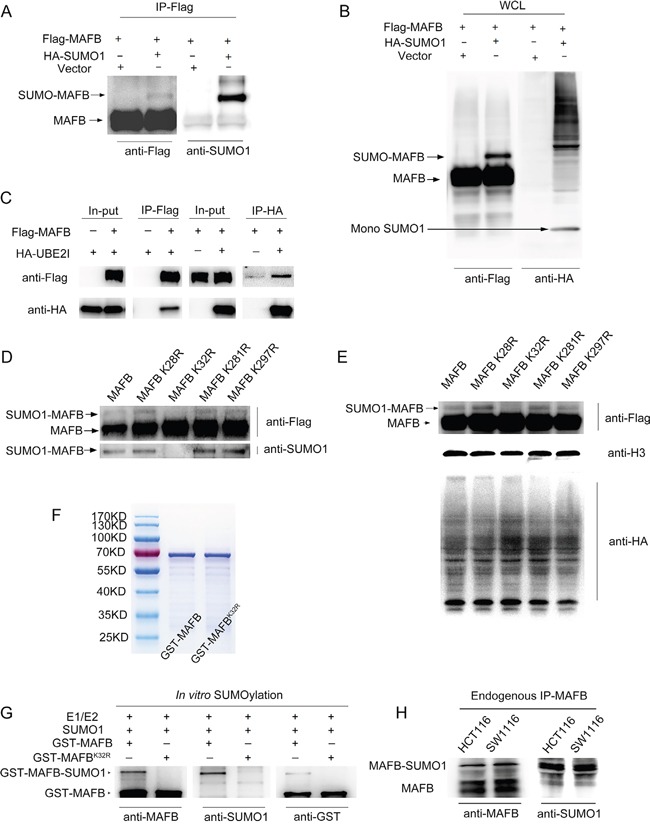
MAFB could be modified by SUMO1 at lysine 32 Flag-MAFB was co-transfected into 293T cells with HA-SUMO1 or mock vector, then immunoprecipitated by anti-Flag Gel and examined by western blotting (WB) for the presence of Flag-MAFB-SUMO1 conjugates **A.** Whole cell lyses (WCL) from **A.** were detected by WB with antibodies against Flag and HA **B.** 293T cells were co-transfected with the indicated vectors (top), and cell lysates were immunoprecipitated with Flag-Gel or HA-Gel **C.** The in-put and precipitates were examined by WB with antibodies against Flag and HA. The indicated four lysine (K) residues were replaced with an arginine (R) through point mutagenesis **D.** Flag-MAFB wild-type and K32R mutants were transfected with HA-SUMO1 vector into 293T cells. Cell lysates were immunoprecipitated by anti-Flag Gel and examined by WB with antibodies against Flag and SUMO1. Whole cell lysates from **D.** were detected by WB with antibodies against HA, Flag and H3 **E.** GST-MAFB and GST-MAFB^K32R^ proteins were purified by GSH-magnetic beads and stained with Coomassie brilliant blue after polyacrylamide gel electrophoresis **F.** SUMOylation assay was performed with the indicated elements (top), and the reactions were analyzed by WB using antibodies against MAFB, SUMO1 and GST tag **G.** WB was performed using antibodies against MAFB (rabbit polyclonal antibody) and SUMO1 to detect MAFB-SUMO1 conjugates immuno-precipitated by goat polyclonal antibody MAFB from SW1116 or HCT116 cell lysates **H.**

SUMOylation is a post translational modifications [18] that plays important roles in cancer progression [19]. As previously reported, murine MafB is modified by SUMO1 [16]; however, whether *Homo sapiens* orthologue MAFB could be SUMOylated remained unclear. To clarify this question, Flag-tagged MAFB with or without HA-tagged SUMO1, and together with E2 conjugating enzyme UBC9, were transfected into HEK293T cells. To protect the conjugation, transfected HEK293T cells were lysed in a non-denaturing buffer supplemented with the SUMO protease inhibitor *N-*ethylmaleimide (NEM) [25]. MAFB proteins were enriched by immunoprecipitation from cell lysates with Flag affinity gel, and immunoblotted with an antibody against Flag or HA. A higher molecular weight form of MAFB was observed in cells with HA-tagged SUMO1. Only one human MAFB SUMOylation band was detectable, rather than the two bands typical of murine MafB SUMOylation [16] (Figure 4A–4B). This result was supported by MAFB and UBC9 Co-IP assay results. UBC9 is the unique E2 enzyme responsible for transferring SUMO to all acceptor proteins [18]. Flag-MAFB and HA-UBC9 were co-transfected into HEK293T cells and then immunoprecipitated with Flag or HA antibodies, respectively. Direct interaction between MAFB and UBC9 was confirmed by western blotting (Figure 4C).

### Human MAFB was SUMOylated by SUMO1 at lysine 32

We determined the candidate SUMO acceptors on MAFB. According to the GPS-SUMO 1.0 software [26] prediction, there are four candidate SUMO sites, K28, K32, K281, and K297, on MAFB fitting the consensus SUMO-substrate motif (ψ-K-X-E, where ψ is a hydrophobic amino acid and X is any amino acid) [27, 28]. Individual Flag-tagged MAFB SUMO-mutants targeting these lysine residues were generated by site-directed mutagenesis. Wild-type MAFB and the mutant plasmids were transfected separately into HEK293T cells with HA-tagged SUMO1. Flag-tagged MAFB was enriched with Flag affinity gel and detected by western blotting with antibodies against Flag and SUMO1. Our results revealed that lysine 32, but not other lysine residues, was responsible for MAFB SUMO1 modification (Figure 4D–4E).

To further confirm SUMO1 modification of MAFB, we performed an *in vitro* SUMOylation assay. GST-MAFB and GST-MAFB^K32R^ proteins were purified via GST pull-down assay and verified by coomassie brilliant blue staining (Figure 4F). Purified proteins were incubated in a reaction containing SUMO E1/E2 enzymes and SUMO1, followed by western blotting to detect the SUMOylation bond using antibodies against MAFB, GST and SUMO1. This bond was observed with wild-type GST-MAFB, but not K32 mutant MAFB, providing further support for MAFB SUMOylation at lysine 32 (Figure 4G).

### SUMOylation of MAFB promotes CRC cell proliferation

To confirm MAFB was also modified by SUMO1 in CRC cells, western blotting was performed using antibodies against MAFB (rabbit polyclonal antibody) and SUMO1 to detect the MAFB-SUMO1 conjugates immunoprecipitated via antibodies against MAFB (goat polyclonal antibody) from SW1116 or HCT116 cell lysates. MAFB was conjugated by SUMO1 in both cell lines (Figure 4H).

**Figure 5:**
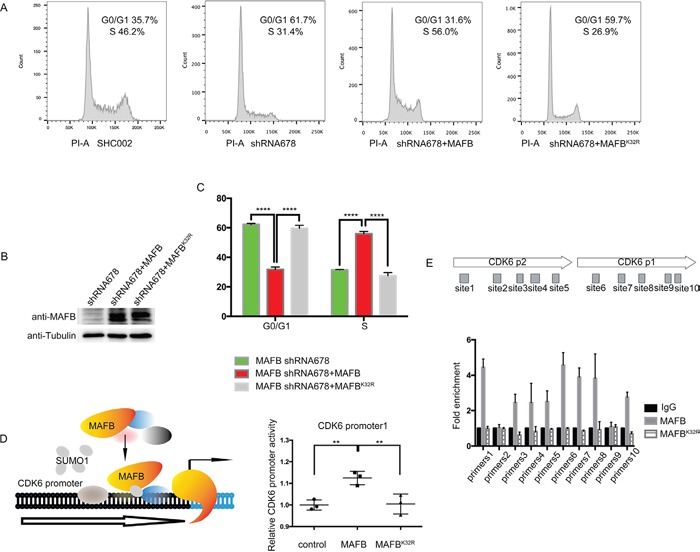
MAFB SUMOylation promotes CRC cell proliferation Representative cell cycle distribution images in the indicated SW1116 cell types as analyzed by flow cytometry **A.** Western blotting (WB) was performed to detect MAFB in the indicated SW1116 cell lines **B.** Percentage of the indicated SW116 cells in G0/G1 or S phase **C.** Schematic of MAFB binding to the human CDK6 promoter regions (left panel), Relative CDK6 promoter activity as analyzed by luciferase assay (right panel), The pRL-SV40 vector and pGL4.15 luciferase reporter vector containing CDK6 promoter regions were co-transfected into 293T with wild-type MAFB and MAFB^K32R^ expression vector or mock vector **D.** ChIP-qPCR analysis of MAFB binding to the CDK6 locus, The MAFB-shRNA678 SW1116 cell line was transfected with Flag-MAFB or Flag-MAFB^K32R^, and ChIP-qPCR was performed with the primer sets indicated in the upper diagram **E.** Data are presented as means ± SEM. **P<0.01; ****P<0.0001.

Because MAFB may regulate the G1/S phase transition and thereby promote CRC cell proliferation, we investigated the functional consequences of MAFB SUMO modification on cell cycle regulation. MAFB shRNA sequence 678 targets the 3′-untranslated region and thus allows simultaneous expression of the vector containing the *MAFB* gene-coding region in shRNA-transfected cells [29, 30]. MAFB shRNA678 knockdown SW1116 cells were infected with retrovirus expressing MAFB or MAFB^K32R^. As shown in Figure 5A, The MAFB deficient cell cycle phenotype was rescued by wild-type MAFB, but not MAFB^K32R^ (Figure 5A–5C & Supplementary Figure S4B–S4D), and MAFB and MAFB^K32R^ overexpression was confirmed by western blotting (Figure 5B & Supplementary Figure S4A). These results indicated that MAFB SUMOylation is crucial to CRC cell cycle regulation.

In this study, the key cell cycle factor CDK6 was shown to be a direct target of MAFB. A luciferase reporter assay was performed to assess the impact of MAFB SUMOylation on CDK6 transcription. Co-transfection of reporter construct with wild-type MAFB promoted CDK6 expression in HEK293T cells; however, the SUMO modification-deficient MAFB^K32R^ lost transactivation activity (Figure 5D). This observation was supported by chromatin immunoprecipitation (ChIP) quantitative PCR results showing that MAFB, but not MAFB^K32R^, bound the CDK6 promoter region (Figure 5E).

### SUMOylated MAFB promotes tumor formation in nude mice

**Figure 6:**
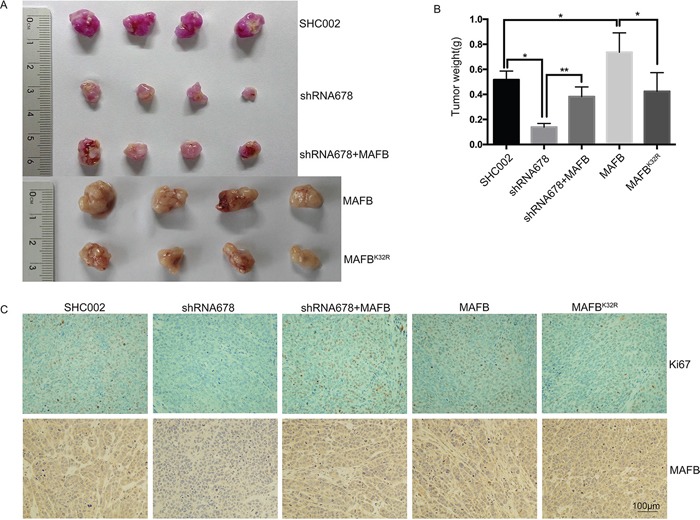
Sumoylated MAFB promotes tumor formation in nude mice SW1116 cell lines were subcutaneously inoculated into nude mice. Representative photographs show dissected xenograft tumors after mice were sacrificed **A.** Tumor weight at the end of the experiment **B.** Data are presented as means ± SEM. *P<0.05; **P<0.01. Representative immunohistochemical staining with anti-Ki-67 and anti-MAFB in xenograft tumors (bars: 100μm) **C.**

Because MAFB promoted CRC cell growth *in vitro*, we assessed its effect *in vivo*. Scramble shRNA (SHC002), MAFB shRNA678, and MAFB shRNA678 plus wild-type MAFB-infected SW1116 cell lines, along with two SW1116 cell lines infected with retrovirus expressing wild-type MAFB or MAFB^K32R^, were subcutaneously inoculated into nude mice to induce tumor xenografts. MAFB shRNA678 plasmid contains mCherry protein expression cassette and lentivirus infection efficiency could be assessed from the pink color of the tumor, and retrovirus infection efficiency was determined via western blotting (Supplementary Figure S5). MAFB knockdown tumors grew much more slowly than scramble shRNA tumors, and this knockdown phenotype could be rescued by wild-type MAFB. Similarly, wild-type MAFB overexpression tumors grew much faster than tumors in the MAFB^K32R^ overexpression group (Figure 6A–6B).

Ki-67 staining revealed reductions in proliferating cells in MAFB knockdown tumors compared with scramble shRNA and wild-type MAFB rescued tumors. MAFB^K32R^ overexpression tumors also exhibited fewer proliferating cells than wild-type MAFB overexpression tumors (Figure 6C). Consistent with our *in vitro* results, TUNEL assays showed no significant apoptotic changes among these groups (data not shown). Taken together, these data suggest that SUMOylated MAFB promotes tumor formation *in vivo*.

## DISCUSSION

MAFB plays important roles in the development and differentiation of various organs, tissues and cells, and MAFB dysregulation has been identified in multiple myeloma [11, 20] and other tumors. However, the role of MAFB in tumor progression remains largely unknown. In this study, we investigated MAFB status in TCGA using the online analysis tool, cBioPortal [31, 32], and found that MAFB was amplified in a majority of cancer types. Notably, altered MAFB levels due to gene amplification, deletion, transcriptional upregulation or truncating mutation occurred in 9% of CRC cases. This finding was confirmed by our clinical CRC sample analysis.

Our study showed that MAFB knockdown inhibited CRC cell proliferation both *in vitro* and *in vivo*. Similar phenomena were also observed in multiple myeloma [33], nasopharyngeal carcinoma [14], and insulinomas [15]. Cell cycle analysis revealed that MAFB knockdown CRC cells were arrested in G1 phase, and levels of cell cycle factors involved in the G1/S transition, including CDK6, CDKN3, CUL1, CUL3 and SKP2, were altered. CDK6 downregulation corresponded with MAFB knockdown, suggesting that MAFB may directly regulate CDK6 expression by binding its promoter. Luciferase reporter and ChIP-qPCR assays confirmed that MAFB regulated CDK6 transcription in both HEK293T and CRC cells. Other groups also observed increased cyclin B1 and D2 levels in MAFB-overexpressing insulinoma cells [15], and this was verified in our experiments (Supplementary Figure S3).

SUMOylation is a key post-translational modification that regulates crucial cellular functions and pathological processes, and is a driver in human tumorigenesis [34]. SUMOylation may affect several aspects of a target protein, including stability, localization and activity [18]. Our results demonstrated that human MAFB, like its mouse orthologue, MafB, could be modified by SUMO1 [16]. However, unlike murine MafB, human MAFB was only modified at lysine 32, rather than both lysine 32 and lysine 297 as in mice. UBC9 is a unique E2 enzyme responsible for transferring SUMO to all acceptor proteins, and can recognize the consensus SUMOylation motif [18]. Our results showed a direct protein-protein interaction between MAFB and UBC9, further confirming human MAFB SUMOylation. Moreover, SUMOylation-deficient MAFB^K32R^ could not rescue the abnormal G0/G1 arrest phenotype induced by MAFB knockdown in CRC cells. Similarly, luciferase reporter assay results showed that that MAFB^K32R^ could not drive CDK6 transcription. ChIP-qPCR assay also showed that wild-type MAFB, but not MAFB^K32R^, bound the CDK6 promoter region. Taken together, these findings indicated that MAFB promoted CRC cell proliferation by regulating the cell cycle, and this regulation was dependent on MAFB SUMOylation at lysine 32.

In conclusion, we reported a new mechanism of CRC pathogenesis. MAFB upregulation was observed in CRC patient tumor tissues, and its regulation on cell cycle was dependent on SUMOylation at lysine 32. MAFB deficiency inhibited CRC progression by dysregulating the expression of cell-cycle factors, thereby arresting cell cycle at G0/G1 phase. MAFB and its SUMOylation process may serve as potential novel therapeutic targets for treatment of CRC.

## MATERIALS AND METHODS

### Data mining and analysis in TCGA

The TCGA database was reproduced here according to TCGA publication guidelines (http://cancergenome.nih.gov/publications/publicationguidelines). We used cBioportal as an online analytical tool to process 631 colorectal adenocarcinoma samples (TCGA, Provisional) [31, 32].

### Cell culture

Human CRC cell lines, HCT116 and SW1116, were purchased from the Shanghai Institutes of Biological Sciences or the American Type Culture Collection (ATCC). Cells were cultured in Roswell Park Memorial Institute 1640 (RPMI1640) medium (Gibico, USA) with 10% fetal bovine serum (FBS) (Gibico, USA). HEK293T cells maintained by our lab were cultured in Dulbecco's Modified Eagle Medium (DMEM) (Gibico, USA). Cells were cultured in a humidity incubator at 37°C with 5% CO_2_.

### Patients and human colorectal tissue specimens

All study procedures were reviewed and approved by the institutional ethics committee of Shanghai Rui Jin Hospital, and all patients were fully informed of the experimental procedures. From 2012 to 2015, 61 sets of gastric tumor and adjacent non-tumorous tissues were obtained from patients who underwent curative surgery at Shanghai Jiao Tong University School of Medicine Affiliated Rui Jin Hospital. Clinical pathological data were collected and pathological tumor staging was determined according to the TNM classification.

### Plasmids construction

MAFB DNA sequences were cloned by PCR using the primers: CCAGCTCTTCTCCGCTCTT (forward) and CGCTCAAGTCAAACAGGTCA (reverse). MAFB coding sequences were cloned into the pFlag CMV4 vector (Sigma, USA) between EcoRI and BamHI restriction enzyme sites, fusing the MAFB protein N-terminus with a Flag tag. MAFB K28R, MAFB K32R, MAFB K281R, and MAFB K297R were generated using PCR-directed mutagenesis, and sequenced. The HA-fused SUMO1 plasmid was cloned into the pPBCAG vector. MAFB and MAFB^K32R^ DNA sequences were amplified by PCR using the primers: cgc*GGATCC*GCCGCGGAGCTGAGCATG (MAFB-BamHI-Forward) and ccg*GAATTC*TCACAGA AAGAACTCGGGAG (MAFB-EcoRI-Reverse), and then inserted into the pGEX-4T-2 vector between BamHI and EcoRI restriction enzyme sites. The full lengths of MAFB and MAFB^K32R^ fused with Flag-Tag were amplified from pCMV-3Tag-1A-MAFB by PCR using the primers: TGGA*AGATCT*CCGCCACCATGGATTACA (MAFB-BglII-Forward) and ccg*GAATTC*TCACAGAA AGAACTCGGGAG (MAFB- EcoRI-Reverse) and then inserted into the MigR1-GFP vector between BglII and EcoRI restriction enzyme sites (italics within primers represent restriction enzyme sites).

MAFB shRNA sequences were obtained from the RNA interference (RNAi) Platform of the BROAD institute [35]. The lentiviral shRNA vectors, including scramble control (SCH002: CAACAAGATGAAGAGCACCAA), MAFB shRNA 678 (TRCN0000017678: GCCTTGTCTTATGGTCAAATT), and MAFB shRNA 679 (TRCN0000017679: GCCCA GTCTTGCAGGTATAAA), were constructed by inserting stem-loop sequences (Shanghai Sunnybio) into pLVX shRNA2 (Clonetech, USA) between BamHI and EcoRI restriction enzyme sites. The ZsGreen coding regions of pLVX shRNA2 were replaced by mCherry coding regions.

### Real-time quantitative PCR (RT-qPCR)

Total RNA was extracted from CRC tissues or cell lines using Trizol reagent (Invitrogen, USA). 1μg of RNA was reverse transcribed to cDNA using the PrimeScript RT reagent Kit with gDNA Eraser (Perfect Real Times) (TaKaRa, Japan). RT-qPCR was performed using SYBR Premix Ex Taq™ II (Perfect Real Time) (TaKaRa, Japan). Primers were as follows: MAFB forward: GACGCAGCTCATTCAGCAG, reverse: CTCGCACTTGACCTTGTAGGC; GAPDH forward: CT CACCGGATGCACCAATGTT, reverse: CGCGTTGC TCACAATGTTCAT; CDKN1B forward: ATCACAAA CCCCTAGAGGGCA, reverse: GGGTCTGTAGTAGA ACTCGGG; CCNE1 forward: AAGGAGCGGGACAC CATGA, reverse: ACGGTCACGTTTGCCTTCC; CDK6 forward: TCTTCATTCACACCGAGTAGTGC, reverse: TGAGGTTAGAGCCATCTGGAAA; CDK4 forward: CTGGTGTTTGAGCATGTAGACC, reverse: GATCC TTGATCGTTTCGGCTG; CCND1 forward: TGGAGC CCGTGAAAAAGAGC, reverse: TCTCCTTCATCTT AGAGGCCAC; CUL3 forward: TGTGGAGAACGT CTACAATTTGG, reverse: GCGCCTCTGTCTACGAC TT; CCNA2 forward: TGGAAAGCAAACAGTAAACAG CC, reverse: GGGCATCTTCACGCTCTATTT; CDC25A forward: GTGAAGGCGCTATTTGGCG, reverse: TGGT TGCTCATAATCACTGCC; SKP2 forward: ATGCCC CAATCTTGTCCATCT, reverse: CACCGACTGAGT GATAGGTGT; CUL2 forward: TATGTGTGGCCTATCC TGAACC, reverse: TGCAAATGCCGAACATGATTTTC; CUL1 forward: AGCCATTGAAAAGTGTGGAGAA, reverse: GCGTCATTGTTGAATGCAGACA; ANAPC2 forward: TATGTTGCGCGGAGTCTTGTT, reverse: GAAGCACCCATACAGACGCTG; CDC34 forward: CCTGAGTGTGATCTCCCTCCT, reverse: TGTCTGTG TACTCCCGATCCT; CDKN3 forward: TCCGGGGCA ATACAGACCAT, reverse: GCAGCTAATTTGTCCCG AAACTC; CCNB1 forward: AATAAGGCGAAGATC AACATGGC, reverse: TTTGTTACCAATGTCCCCAAG AG; CCND2 forward: TTTGCCATGTACCCACCGTC, reverse: AGGGCATCACAAGTGAGCG. All reactions were performed on an Applied Biosystems 7500 Real-time PCR system (Applied Biosystems, USA). Relative mRNA levels were normalized against GAPDH and calculated using the 2^−ΔΔCt^ method.

### Cell proliferation assay

Cell growth was analyzed using a WST-8 Cell Counting Kit-8 (Dojindo, Japan). 2×10^3^ CRC cells were seeded in each well of a 96-well plate with 100μL RPMI1640 medium containing 10% bovine serum. CCK-8 solution (10μL) was added to each well and plates were incubated at 37°C for 120 min. Absorbance at 450nm was measured using a spectrophotometer. Results were plotted as means ± SD of three independent experiments.

### Cell apoptosis assay

Cell apoptosis was assayed using the Annexin V-FITC apoptosis detecting kit (Biotool, China). Cells were washed twice with cold PBS and resuspended in 100 μL 1× binding buffer at 5×10^5^ cell/mL. Cells were stained with Annexin V-FITC/APC and propidium iodide (PI), and analyzed by flow cytometry. Experiments were repeated three times.

### Cell cycle analysis

1×10^6^ cells were collected, washed twice with cold PBS, and fixed with cold ethanol before being stored at −20°C for at least 24 h. Fixed cells were washed and resuspended in 400μl PBS containing 50μg/ml Rnase A, and then incubated for 30 min at 37°C. Cells were analyzed for DNA content by flow cytometry after incubation with 50μl 1mg/ml PI for 15 min. For each sample, 10000 events were acquired, and cell cycle distribution was determined using cell cycle analysis software (Flowjo, USA). “Watson Pragmatic” was used to analyze the cell cycle. Experiments were performed three times independently.

### SUMOylation assay

MAFB SUMOylation was analyzed in HEK293T cells via co-immunoprecipitation (Co-IP) with anti-Flag Affinity Gel (Sigma, USA) and anti-HA Affinity Gel (Biotool, China). The cell-free SUMOylation assay was performed as follows: MAFB protein and MAFB 32 lysine-to-arginine mutation protein were expressed independently in *E.coli* BL21 cells, then purified by GST-pull down. SUMOylation was performed as previously described [36]. Briefly, 10× SUMOylation buffer was prepared, containing 500mM Tris-HCl (pH7.6), 500mM KCl, 50mM MgCl_2_, 10mM DTT, and 10mM ATP. Each SUMOylation reaction mixture, prepared on ice, contained 1μl 10× SUMOylation buffer, 0.4 unit creatine phosphokinase, 10mM creatine phosphate, 0.6 unit inorganic pyrophosphatase, 100ng purified SAE1/SAE2 heterodimer (E1) (Boston biochemistry, USA), 400ng purified ubc9 (E2) (Boston biochemistry, USA), and 1μg purified SUMO1 (Boston biochemistry, USA), with H_2_O to 10μl and then incubate at 37°C for 1 h. Terminate the reaction by adding 12 μl 2×SDS-PAGE loading buffer, then boil the samples for 5 min and separate on SDS-PAGE gel.

### Luciferase reporter assay

The human CDK6 promoter1 region (Homo sapiens chromosome 7, GRCh38.p7 Primary Assembly, range: 92833899 to 92835931) was amplified by PCR using the primers: TCTA*GCTAGC*ACAGAAAAGTGCACGCAGC (P1 forward) and ACCC*AAGCTT*CTTCGCGCGGAGAGGTTG (P1 reverse). The promoter2 region (Homo sapiens chromosome 7, GRCh38.p7 Primary Assembly, range: 92836583 to 92838620) was amplified by PCR using the primers: CGG*GGTACC*CTGATCTAGCAATGGTTGTT (P2 forward) and TCTA*GCTAGC*GGAGTTACCAGCACTCTC (P2 reverse) (italics within primers represent restriction enzyme sites). The CDK6 promoter1 and promoter2 regions were cloned into the NheI/HindIII and KpnI/NheI sites, respectively, of the pGL4.15 luciferase reporter vector (Promega, USA). All constructs were verified by sequencing.

293T cells were plated into 48-well plates and transfected with pGL4.15 CDK6 P1 or pGL4.15 CDK6 P2 (100ng per well) and pRL SV40 vector (Promega, Madison, USA) (100ng per well), along with the wild-type MAFB and MAFB^K32R^ expression vector (300ng per well). Cells were harvested and lysed for luciferase assay 24 h post-transfection. *Renilla* and firefly luciferase activities were measured using the Dual-luciferase Reporter Assay System (Promega, USA). Values were normalized to *Renilla* luciferase activity. Experiments were repeated three times.

### Chromatin immunoprecipitation (ChIP) assay

ChIP assay was performed using the EZ-CHIP^TM^ chromatin immunoprecipitation kit (MercK Millipore, Germany). Genomic DNA was cross-linked to proteins by adding formaldehyde to culture medium (final concentration: 1%). Cells were collected and resuspended in lysis buffer containing Protease Inhibitor Cocktail II. Lysate was sonicated to reduce DNA to approximately 200–1000 base pairs in length. The Flag anti-body (CST, USA) and protein G agarose were used to precipitate the chromatin complex. Finally, DNA was collected, and RT-qPCR was performed to analyze the precipitates. Primers for the CDK6 promoter containing putative MAFB binding sites were as follows: sense: tagccatgatgtgctgcttc, antisense: cacaagtgggaatcctccat (for site 1); sense: tcaaacagaattttgccaggt, antisense: ttggt gttctccccaaaatc (for site 2); sense: cgttagtgaaccctcagcag, antisense: tttggaaattgtcaggcactc (for site 3); sense: gca tcctgccaaactttttc, antisense: acggatgggaaaaacacaaa (for site 4); sense: ctagcccccgactcgtaga, antisense: agtggc aagaaagtgtgcaa (for site 5); sense: agaggcgcatgaaggattt, antisense: ttcaacatttccgcattcct (for site 6); sense: ctccc cggctacctgtgt, antisense: gagcagagcttctggcactc (for site 7); sense: tactctggcgctttgttgtg, antisense: ccgctgtaggtagcagaggt (for site 8); sense: ggtcttcccaaggtttccac, antisense: cgactc gcatacacaaatgg (for site 9); sense: agatgtctgaccaccccttct, antisense: aaggactgtgggtccatcc (for site 10).

### Protein extraction and western blotting

CRC cells were washed twice with cold PBS and then lysed on ice with RIPA lysis Buffer (Beyotime, China) containing protease inhibitors (Roche, Switzerland). Protein concentrations were measured via Braford reagent (Sangon Biotech, China). Equal amounts of denatured protein from all groups were run through a 5%-20% gel via SDS-PAGE and then transferred onto Immobilon Western blotting transfer membranes (Millipore, USA). Membranes were blocked for 2 h in Tris buffered saline with 0.1% tween 20 (TBST) containing 3% bovine serum albumin (Sangon Biotech, China), and then incubated overnight at 4°C with primary antibody against MAFB (Abcam, UK), GAPDH (Abmart, China), Tubulin (Proteintech, USA), SUMO1 (CST, USA), H3 (CST, USA), HA tag (Abmart, China), or Flag tag (Sigma, USA) at the recommended dilutions. After three washes with TBST, membranes were incubated with the corresponding horseradish peroxidase-conjugated secondary antibodies at room temperature for 2 h and then detected with Chemiluminescent HRP substrates (Millipore, USA).

### Virus preparation and xenograft model

Lentivirus particles were harvested 48 h after pLVX-shRNA2 MAFB shRNA678, pLVX-shRNA2 MAFB shRNA679 and pLVX-shRNA SHC002 were transfected together with helper plasmids (pMD2.G and psPAX2) into 293T lentiviral packaging cells using the Calcium Phosphate Cell Transfection Kit (Beyotime, China). Retrovirus particles were harvested 48 h after transfecting MigR1-GFP MAFB and MigR1-GFP MAFB^K32R^ with helper plasmids (VSVG and Gag pol) into 293T cells using the Calcium Phosphate Cell transfection Kit. Target cells were infected with lentivirus particles plus 8μg/mL polybrene (Sigma, USA).

Animal experiments were performed according to the protocol approved by the institutional animal experimentation committee. Female BALB/c-nude mice aged 4 weeks were used for the human tumor xenograft model. Twenty mice were randomized into five equal groups: SW1116 scramble, SW1116 MAFB shRNA678, SW1116 MAFB shRNA 678 + MAFB rescue, SW1116 MAFB overexpression, and SW1116 MAFB^K32R^ overexpression. Mice were subcutaneously inoculated with 2x10^6^ SW1116 cells. Three weeks later, all mice were sacrificed and tumors were excised and weighed. At least four tumors were collected from each group for further analyses.

### Immunohistochemical analyses

Paraffin-embedded xenograft tumors were cut into 5-μm sections and prepared for staining. Primary antibodies against Ki-67 (Santa Cruz, USA) and *MAFB* (Abcam, UK) were added to slides at the recommended dilutions, and incubated overnight at 4°C. Slides were then washed with PBS and incubated for 2 h at room temperature using the Envision kit (DAKO, USA). Two pathologists blinded from any patient data independently examined the cellular location of MAFB and compared staining between tumor and normal tissues for each case. The percentage of positive cells was classified into five grades (percentage scores): <10% (grade 0), 10–25% (grade 1), >25–50% (grade 2), >50–75% (grade 3), and >75% (grade 4). Immunohistochemical staining intensity was graded as follows: 0 (no staining), 1 (bright yellow), 2 (orange), or 3 (brown). Total scores of ≤2, >2–5, and ≥6 were defined as negative, weak positive, and strong positive staining, respectively [37].

### Statistical analysis

All statistical analyses were performed using Prism 6 or SPSS software. Data are shown as means ± SD. Student's t test was used for comparisons of two groups. Comparisons of multiple groups to a control were performed via one-way analysis of variance (ANOVA) in Prism 6. Correlations between MAFB expression in CRC tissues and clinicopathological parameters were analyzed by chi-square test (χ2 test) in SPSS. *P*<0.05 was considered significant.

## SUPPLEMENTARY FIGURES


