# Supplementary material for: SUMOylated MAFB promotes colorectal cancer tumorigenesis
Source: Oncotarget. 2016 Nov 5;7(50):83488–501. doi: 10.18632/oncotarget.13129 (PMC5347783; doi:10.18632/oncotarget.13129)
Supplement: Supplementary file 1 [file oncotarget-07-83488-s001.pdf]

SUMOylated MAFB promotes colorectal cancer tumorigenesis

SUPPLEMENTARY FIGURES

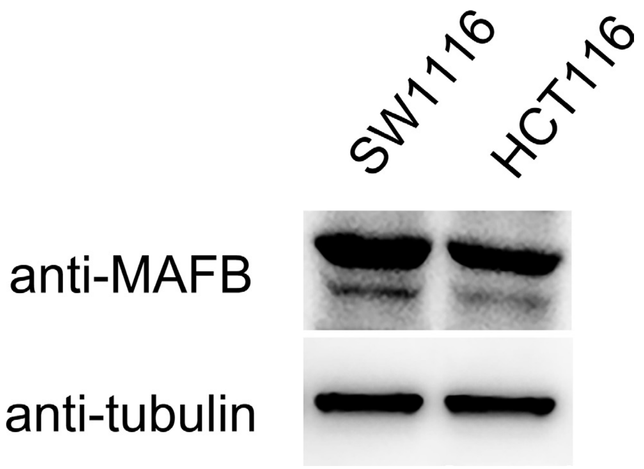

Supplementary Figure S1: Endogenous MAFB expression patterns in CRC cell lines. SW1116 and HCT116 cells were harvested and lysed with Ripa buffer, and MAFB was detected by western blotting.

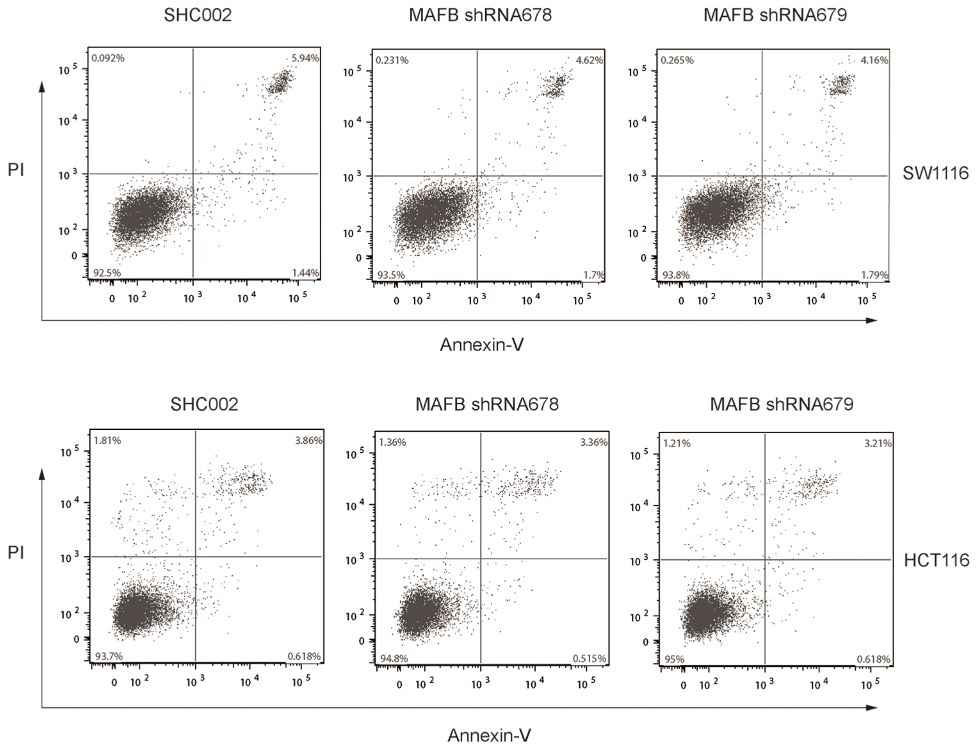

Supplementary Figure S2: Apoptosis in two CRC cell lines after MAFB knockdown. Cells were harvested and stained with annexin-V and PI, and analyzed via flow cytometry.

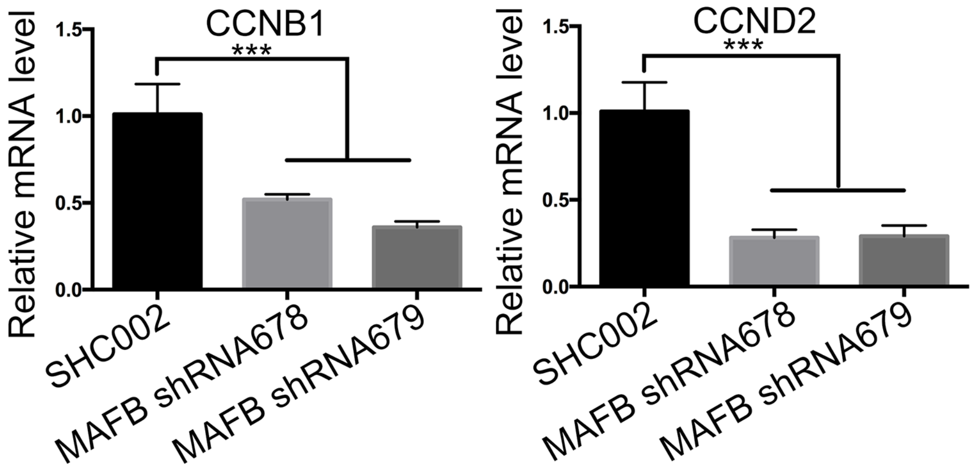

Supplementary Figure S3: The previous reported cell cycle factors regulated by MAFB. RNA was extracted from SW1116 cells transfected with scramble shRNA (SHC002), MAFB shRNA678 or MAFB shRNA679 and the expression level of CCNB1 and CCND2 were analyzed via RT-qPCR.

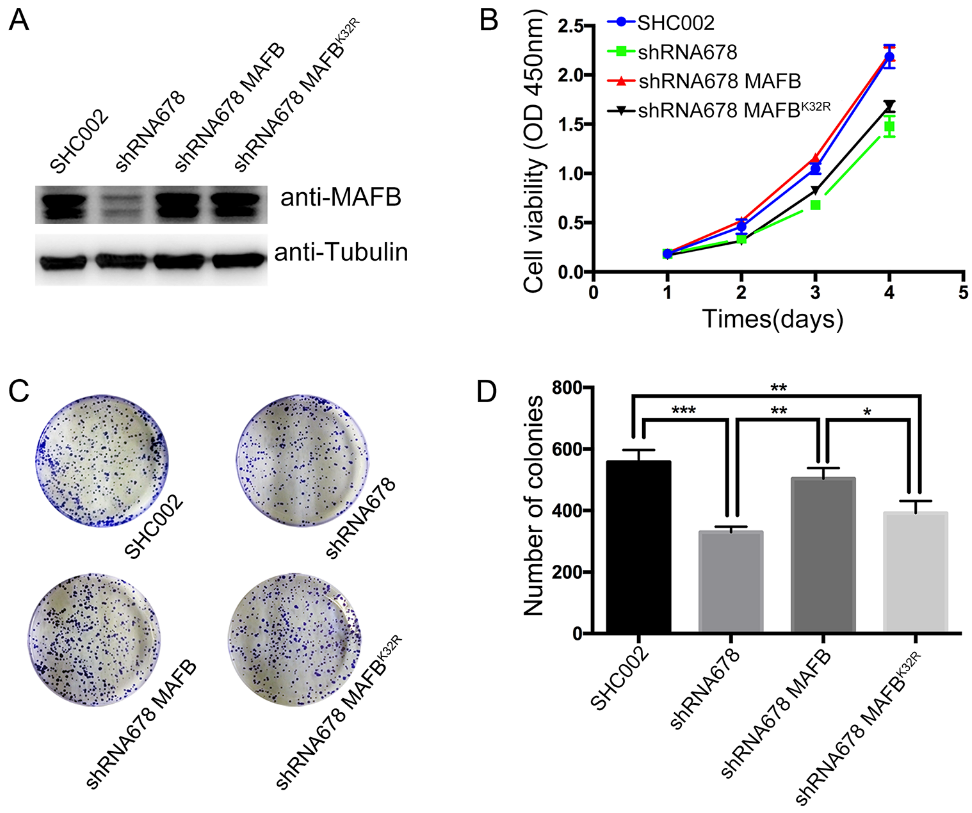

Supplementary Figure S4: MAFB overexpression promoted SW1116 cell proliferation. MAFB was detected by western blotting **A.** SHC002: cells expressing control shRNA SHC002; shRNA678: cells expressing MAFB shRNA 678; shRNA678 MAFB/ MAFB^{K32R}: cells expressing shRNA678 and MAFB or MAFB^{K32R} overexpression vector. Cell viability was analyzed via CCK8 assay **B.** Cell proliferation was assessed via colony formation assay **C.** Data are presented as means ± SEM. **D.** Colonies were counted and analyzed. *P<0.05; **P<0.01; ***P<0.001.

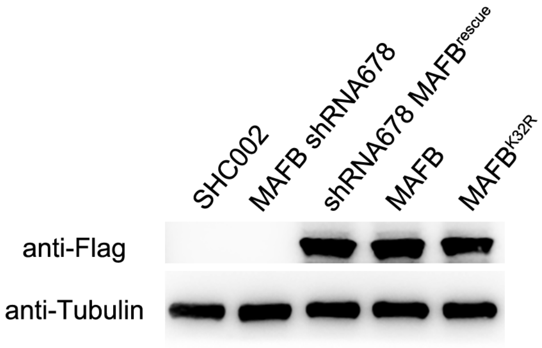

Supplementary Figure S5: Retrovirus infection efficiency. Flag-fused MAFB expression by retrovirus-infected cells used in xenograft experiments was detected by western blotting using a Flag antibody.
